# Close of the Volume

**Published:** 1873-03

**Authors:** 


### EDITORIAL AND MISCELLANEOUS.

### CLOSE OF THE VOLUME.

With this number the tenth volume of the ATLANTA MEDICAL AND SURGICAL JOURNAL closes. While there is much to regret, in our failure to furnish such a journal as we should have desired, still we have had most flattering evidences of more than satisfaction upon the part of its patrons. In truth, with doubtless a considerable mass of material not possessing a very large amount of originality or special merit, the JOURNAL has contained no inconsiderable number of contributions of the *highest* order from a number of eminent medical men, who have kindly sustained us in the endeavor to do something for Southern medical literature.

Our publishers promise to issue the JOURNAL with more regularity, and we hope that no further ground of complaint will exist in the future on this account.

In addition to large expectations for the coming volume from many of our regular corps of contributors, we hope to add many others; and to the already interesting and valuable correspondence from Europe, we are promised an addition from an intelligent medical gentlemen (W. A. Mitchell, M.D., of Glennville, Alabama,) from whom we published some highly entertaining and profitable correspondence from New York in the January number.

And last, though not of the smallest importance, we shall add one or more of our most valued contributors to the editorial corps, their labors in this connection commencing with the next number, being the first of the eleventh volume. These gentlemen will be no mere "figure-heads," but are well qualified, not only in unusual attainments in medical science for the conduct of a medical journal, but are thorough and laborious workers, and we have no hesitation in promising from this source a very large increase of interest and value in the succeeding volume. The gentlemen referred to are well known to the readers of this journal, and will speak for themselves in the succeeding issues.

Besides a large increase of editorial upon matters of current interest, involving the whole scope of our science, it is the intention to furnish our readers in the future with the substance of all important practical contributions to every department of medical science throughout the world, and to this end special assignments of labor will be made to each member of the editorial corps, and with the ample facilities now and to be in our possession, we are confident that nothing of real value or importance will escape attention.

